# The Effect of TAVR on Left Ventricular and Left Atrial Mechanics in Patients with Aortic Stenosis

**DOI:** 10.3390/jcdd9020035

**Published:** 2022-01-21

**Authors:** Malcolm Anastasius, Richard Ro, Michael Gavalas, Neil Patel, Francesca Romana Prandi, Gilbert H. L. Tang, Parasuram Krishnamoorthy, Samin K. Sharma, Annapoorna Kini, Stamatios Lerakis

**Affiliations:** 1 Division of Cardiology, Icahn School of Medicine at Mount Sinai, Mount Sinai Hospital, 1 Gustave L. Levy Place, New York, NY 10029, USA; malcolm.anastasius@mountsinai.org (M.A.); richjro@gmail.com (R.R.); michael.gavalas@gmail.com (M.G.); neil.patel3@mountsinai.org (N.P.); Francesaromanaprandi@gmail.com (F.R.P.); parasuram.melarcode-krishnamoorthy@mountsinai.org (P.K.); samin.sharma@mountsinai.org (S.K.S.); annapoorna.kini@mountsinai.org (A.K.); 2 Department of Cardiovascular Surgery, Icahn School of Medicine at Mount Sinai, Mount Sinai Hospital, New York, NY 10029, USA; gilbert.tang@mountsinai.org

**Keywords:** TAVR, left atrial strain, left ventricular strain

## Abstract

**Background.** Measures of adverse cardiac remodeling, left ventricular global longitudinal strain (LVGLS) and left atrial (LA) phasic function, are predictive of cardiac events in patients with severe aortic stenosis (AS). How these parameters of cardiac function change following TAVR requires further investigation. **Methods.** A number of 109 consecutive patients with symptomatic severe AS who were seen in the heart valve clinic between 2014 and 2019 for TAVR were included. All patients underwent echocardiographic assessment prior to and 30 days following TAVR, with LVGLS and LA phasic function evaluation using 2D speckle-tracking echocardiography. Heart failure hospitalization, and death were assessed at 12 months. **Results.** The mean age of the study cohort was 81 ± 7.3 years. Following TAVR, there was a significant reduction in NYHA class III/IV symptoms [89 (82%) vs. 12 (11%), *p* < 0.01], and median mean aortic valve gradient [44 mmHg (16) vs. 9 mmHg (7), *p* < 0.01]. There was no significant change in the median LVEF [62% (13) vs. 62% (6.0), *p* = 0.2]; however, the LVGLS significantly increased following TAVR [15 ± 3.5% vs. 18 ± 3.3%, *p* < 0.01]. The median LA reservoir, conduit and contractile function significantly improved following TAVR [22.0% (14.0) vs. 18.0% (14.0) *p* < 0.01, 8.9% (5.4) vs. 7.8% (4.8) *p* < 0.01, 12% (11.0) vs. 9.6% (11.0) *p* < 0.01, respectively]. The incidence of death or heart failure hospitalization at 12 months was low, and occurred in eight patients (7.3%). **Conclusions.** TAVR results in significant short-term reverse LV and LA remodeling, as shown by improvement in LV GLS and all three components of LA phasic function, despite no change in the LVEF. The findings indicate the possible utility of strain imaging for the assessment of global LV and LA function following TAVR.

## 1. Introduction

Transcatheter aortic valve replacement (TAVR) for aortic stenosis (AS) is rapidly advancing, with several devices now approved for routine clinical care [1]. Any patient population with severe AS presenting for TAVR is heterogeneous, with varying degrees of left ventricular (LV) and left atrial pathology [2]. Aortic stenosis, through an increase in afterload, results in compensatory left ventricular hypertrophy that, in turn, leads to reduced LV compliance, diastolic dysfunction, elevated end-diastolic pressures, and in some instances, systolic dysfunction [2]. Amongst AS patients, a measure of global LV systolic function and LV global longitudinal strain is predictive of mortality, independent of LVEF [3]. In addition, severe AS results in impaired LA function [4]. Each of the three phases of LA function in AS have been variably investigated, these include: firstly, reservoir function during ventricular systole and isovolumic relaxation; secondly, conduit function in early and mid-diastole when blood flows passively through the left atrium and into the left ventricle; thirdly, contractile function in late diastole to end LV filling [4]. 

Whilst prior studies have separately shown improvement in LV and LA mechanics following TAVR [5], both LV global longitudinal strain (GLS) and LA strain in each of the three phases have not been concurrently evaluated in a large cohort of patients undergoing TAVR with contemporary devices. There is emerging evidence showing an association between improvements in LV GLS following TAVR and lower longer-term mortality [6]; however, the relationship between changes in LA strain after TAVR and clinical outcome is less well known. In addition, there is limited description of changes in both LV GLS and LA strain according to baseline aortic stenosis flow-gradient patterns. We sought to investigate a consecutive contemporary cohort of AS patients in regard to global LV and phasic LA function, using 2D speckle-tracking echocardiography, prior to and following afterload reduction with TAVR.

## 2. Methods

### 2.1. Study Design and Patient Population

Patients referred for potential TAVR for severe aortic stenosis to the Heart Valve Program at Mount Sinai Hospital (New York, NY, USA) were prospectively recruited between 2014 and 2019. Inclusion criteria were severe aortic stenosis and availability of echocardiographic imaging, which is part of the routine evaluation pathway. Baseline clinical demographic data was collected for all patients. Follow-up comprised of clinical evaluation and transthoracic echocardiography. The Kansas City Cardiomyopathy (KCCQ) symptom score was obtained for patients at baseline and at 30 days following TAVR. Patients were followed-up for 12 months following TAVR for monitoring of clinical outcomes of heart failure hospitalization, or death. 

### 2.2. Echocardiography

All echocardiographic studies were performed as part of routine clinical care (predominantly Phillips, EPIQ) at baseline and then at 30 days following TAVR. Cardiac chamber volumes and LVEF were assessed according to the American Society of Echocardiography (ASE) guidelines [7]. AS severity grading and post-TAVR evaluation were performed using an integrated approach according to the ASE guidelines [8]. 

The left ventricular and left atrial strain analysis using 2D speckle-tracking echocardiography was performed blinded to all clinical data, at baseline and 30 days post-TAVR using Qlab 13.0 (Phillips, Best, the Netherlands). For LV analysis, standard 2D images of the apical 4-chamber, 2-chamber and long-axis were obtained at between 60–80 frames/s. A semi-automated algorithm was used to track the LV myocardial wall, which was divided into 18 segments to obtain the global peak longitudinal strain. For LA analysis, non-foreshortened apical 4-chamber and 2-chamber views were used. Images were also acquired at 60–80 frames/s. LA strain was determined during 3 phases of the LA cycle, reservoir, conduit and contractile phases. The LA endocardial border was manually traced, generating a region of interest (ROI). Following segmental tracking and manual adjustment of the ROI, the software package generated longitudinal strain curves. LA strain was measured with the zero-reference standard at end-diastole and at the onset of atrial contraction. Reservoir function (εR) (strain value at mitral valve opening—ventricular end-diastole), LA conduit function (strain at onset of atrial contraction—strain at mitral valve opening) and contractile function (εCT) (strain at ventricular end-diastole—strain at onset of atrial contraction) were then derived (Figure). 

All patients included in the study had severe AS (AVA ≤ 1.0 cm^2^); LVGLS and LA phasic function at baseline and at 30 days post-TAVR were compared across the 4 groups of severe AS, classified according to the baseline flow-gradient pattern described in the American College of Cardiology/American Heart Association guidelines [9]:

1. High-gradient AS, mean gradient (MG) ≥ 40 mmHg;
2. High-gradient, low EF AS: MG > 40 mmHg, LVEF < 50%;
3. Classical low flow, low-gradient AS: MG < 40 mmHg and LVEF < 50%;
4. Paradoxical low flow, low-gradient AS: MG < 40 mmHg, LVEF ≥ 50% and Svi < 35 mL/m^2^.

### 2.3. Statistics

Continuous variables are expressed as mean ± SD and categorical variables as numbers of events with percentages. Student’s *t*-test, or an ANOVA with Tukey–Kramer or the Bonferroni method were used for continuous variables. For non-normally distributed data, the Mann–Whitney test was used for unpaired data analysis, and the Wilcoxon test for paired data analysis. Chi-square tests were used for categorical variables. Linear regression analysis was performed to determine univariate predictors of change in KCCQ symptom score > 27.6 following TAVR. A 2-sided *p*-value < 0.05 was considered significant for all tests. All statistical analyses were performed using IBM SPSS (Version 22, Armonk, NY, USA: IBM Corp). 

## 3. Results

### 3.1. Patient Characteristics

Between 2014 and 2019, 109 patients with severe aortic stenosis underwent assessment of LA function prior to and following TAVR. The clinical characteristics of the study subjects are shown in Table 1. The mean age of the cohort was 81 ± 7.3, and 49% were male. A history of diabetes mellitus, hypertension and myocardial infarction were present in 34%, 96% and 20%, respectively. Atrial fibrillation or flutter history was present in 33%. A sizeable population of the cohort (82%) had NYHA class III/IV symptoms (Table 1). The median LVEF at baseline was 62.0% (13.0), with a reduced mean LVGLS of 15.0% (3.5). The majority had normal high-gradient aortic stenosis (71%), with classical and paradoxical LF LG AS seen in 8.3% and 11%, respectively. The median aortic valve area was 0.7 cm^2^ (0.2). The self-expanding Medtronic valve was the more commonly implanted valve (58%), with the majority being either 26 mm or 29 mm in size (Table 2). 

### 3.2. Clinical and Echocardiographic Findings Pre- and Post-TAVR

Following TAVR, there was a significant reduction in NYHA class III/IV symptoms [89 (82%) vs. 12 (11%), *p* < 0.0001], median mean aortic valve gradient [44 mmHg (16) vs. 9 mmHg (7), *p* < 0.0001] and improvement in the KCCQ12 score [34 (25) vs. 79 (33), *p* < 0.0001]. There was no significant change in the median LVEF [62% (13) vs. 62% (6.0), *p* = 0.2]; however, the LVGLS significantly increased following TAVR [15 (3.5) vs. 18 (3.3), *p* < 0.0001] (Table 3). 

**Figure 1:**
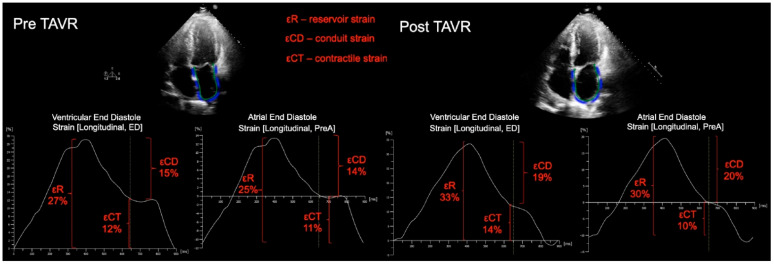
Representative example of LA strain measured for a patient prior to, and following TAVR, showing improvement in LA phasic function. For LA strain analysis, apical 4-chamber and 2-chamber views were used (4-chamber views only shown); the LA endocardial border was manually traced, generating a region of interest (ROI). Following segmental tracking and manual adjustment of the ROI, the software package generated longitudinal strain curves. LA strain was measured with the zero-reference standard at end-diastole and at the onset of atrial contraction. Reservoir function (εR), (strain value at mitral valve opening—ventricular end-diastole), LA conduit function (strain at onset of atrial contraction—strain at mitral valve opening) and contractile function (εCT) (strain at ventricular end-diastole—strain at onset of atrial contraction) were then derived.

There was no significant change in left atrial volume index following TAVR (46 (25) vs. 47 mL/m^2^ (19), *p* = 0.48). However, when using the ventricular cycle, and ventricular end-diastole as the zero reference, the LA εR, conduit and εCT function significantly improved following TAVR [22.0% (14.0) vs. 18.0% (14.0) *p* < 0.0001, 8.9% (5.4) vs. 7.8% (4.8) *p* = 0.002, 12% (11.0) vs. 9.6% (11.0) *p* = 0.003, respectively]. Likewise, when the atrial cycle was used, with atrial contraction as the zero reference, the LA strain increased across all three phases [19.0% (11.0) vs. 17.0% (11.0) *p* < 0.0001, 8.2% (4.8) vs. 7.0% (4.0) *p* = 0.01, 11.0% (9.1) vs. 8.8% (9.2) *p* = 0.004, respectively) (Table 3) (Figure 1). 

Whilst there was a more significant reduction in mean aortic valve gradient following implantation of the self-expanding valve (Medtronic Evolut Pro/Pro+), compared with the balloon-expandable valve ((Edwards Sapien 3 Ultra) (−83% (12%), −72% (18%), *p* < 0.0001), there was no difference in LA function when comparing the two prosthetic valve types (Supplementary Table S1). 

### 3.3. Echocardiographic Results According to LVEF-Flow-Gradient Pattern

When patients were divided according to baseline LVEF-flow-gradient patterns, those with classical LF LG and low-EF high-gradient AS demonstrated a significant rise in LVEF compared with patients with normal high-gradient AS (+17% (30) vs. 0% (16), *p* = 0.01 and +31% (78) vs. 0% (16), *p* = 0.02 respectively). There was no significant change in LA strain during the reservoir, conduit and contractile phases when comparing patients across all LVEF-flow-gradient patterns (Table 4).

### 3.4. Clinical Events, Symptoms and Echocardiographic Parameters Following TAVR

At 12 months, 8 patients had either been hospitalized for heart failure or suffered death. The majority of the clinical events were due to heart failure hospitalization, with only 1 death at 12 months. Baseline LA strain, LAVI, LV GLS or LVEF, nor the median percentage change in these parameters at 30 days post TAVR were associated with heart failure hospitalization or death at 12 months (*p* > 0.05 for all) (Table 5 and Table 6). 

## 4. Discussion

We evaluated left ventricular and left atrial mechanics prior to and following TAVR, using 2D speckle-tracking echocardiographic strain measurements. Following TAVI, our patients showed a significant reduction in aortic transvalvular gradients. Concurrently, there was a significant improvement in LV global longitudinal strain, LA εR, conduit and εCT function, 3 months following TAVR, whereas the LVEF did not significantly improve. These findings emphasize the importance of strain imaging, as compared with LVEF evaluation for the assessment of global LV systolic and phasic LA function, in patients undergoing TAVR. 

Despite the mean LVEF of the AS cohort being in the normal range, the LVGLS was reduced. 2D speckle-tracking LV GLS provides a more sensitive measure of myocardial systolic function that LVEF [3]. In addition, LVGLS is independently predictive of mortality, irrespective of LVEF [3]. Following TAVR, there was a significant improvement in LVGLS, despite no significant change in LVEF for the total cohort. 

Paralleling the improvement in LVGLS, there was also significant enhancement in phasic LA function following TAVR. Whilst prior studies have shown a reduction in the different phases of LA function in AS, and improvement in LA function following TAVR [5], the present study, firstly, evaluated changes in LA function across a larger patient population that underwent TAVR with more contemporary devices and, secondly, investigated patterns of reverse remodeling and its relationship to symptoms. Furthermore, we have shown improvement in LA εR, conduit and εCT function, whereas a prior study evaluated εR and εCT function only [5]. Left atrial function is important for maintaining optimal cardiac output, in the setting of the impaired LV relaxation and reduced LV compliance observed in AS [4]. Poor LA function in AS may thus predispose patients to clinical deterioration and increase the risk of developing of atrial fibrillation [4]. It is also predictive of major adverse cardiac events [4]. Given that LA strain is load dependent and influenced by LV function, it has been suggested that LA contractile (pump) function is an ideal measure for LA function assessment, as opposed to reservoir function [10]. Reservoir and conduit function are related to LV filling pressures and LV relaxation, and improvements in these following TAVR may be explained by the improvement in LV function, rather than reverse remodeling [11]. However, in the present study, LA contractile function improved following TAVR, suggestive of reverse LA cavity remodeling. 

Additionally, we have shown that LA volumetric assessment identified no change in LA volumes following TAVR. In fact, a prior study demonstrated that LA passive function and conduit function reduction in AS was not related to LA passive volume or LA conduit volume. Whilst a prior study showed improvement in LA volume following surgical AVR, this was in a younger, lower-risk cohort [12]. The different finding in the present study may be explained by more advanced remodeling at baseline, in our relatively higher-risk cohort.

Evaluation of LA function following TAVR according to baseline LVEF flow-gradient patterns has not been previously described. We found no change in LV GLS and LA function when stratified according to LVEF-flow-gradient patterns. A prior study showed improvement in LV GLS in LFLG AS with reduced and preserved LVEF at a later time point of 6 months post-TAVR [13], an interval period where a greater extent of LV cavity reverse remodeling occurred. Moreover, in the present study, there was no improvement in LA function when the patient cohort was stratified according to AS flow-gradient patterns. A change in LA function may not have been seen, as factors known to independently influence LA phasic function, such as mitral valve disease, atrial fibrillation, and LV restrictive physiology, were not controlled for the analysis. In addition, the sample size of patients without a normal high-gradient AS was small. 

The lack of association between baseline LA strain, LV GLS and the median percentage change in these parameters at 30 days post-TAVR, with heart failure hospitalization or death at 12 months, is likely reflective of the low clinical event rate. Notably, a lower reduction in left atrial strain following TAVR has been shown to be independently predictive of cardiovascular death and hospitalization for heart failure [14].

### Study Limitations

There are several limitations of the present study that should be addressed. This was a single center study of a small patient population. Patients with atrial fibrillation were not excluded from the study, given this arrhythmia is common amongst AS patients. The absence of atrial contraction in these patients does effect our results. We sought to evaluate LA phasic function across different AS LVEF flow-gradient subtypes; however, our findings were limited by a small number of patients without classical high-gradient AS. The low incidence of clinical events in the follow-up period likely contributed to the lack of association between LA strain and LV GLS with heart failure hospitalization or death.

## 5. Conclusions

We have shown, in a contemporary AS population, that TAVR results in significant short-term reverse LV and LA remodeling, as shown by the improvement in LV GLS and all three components of LA phasic function, despite no changes in the LVEF. The findings indicate the possible utility of strain imaging for the assessment of global LV and LA function following TAVR. Future studies, with a larger study population and a longer-term follow-up, are required.

## Figures and Tables

**Table 1:** Baseline Patient Demographics.

Group Characteristics	n (%)
**Total patients**	109 (100%)
**Mean Age (SD)**	81 (7.3)
**Male Sex**	53 (49%)
**Ethnicity**	
White	78 (72%)
Black	10 (9.2%)
Asian	5 (4.6%)
Other/unspecified	16 (15%)
**Diabetes Mellitus**	37 (34%)
Insulin dependent	17 (16%)
Oral hyoglycemics	13 (12%)
Diet and Lifestyle modification	7 (6.4%)
**Hypertension**	105 (96%)
**Tobacco Smoker**	5 (4.6%)
**Chronic Lung Disease**	33 (30%)
Mild	18 (17%)
Moderate	8 (7.3%)
Severe	7 (6.4%)
**Peripheral Artery Disease**	10 (9.2%)
**Prior Stroke**	14 (13%)
**ESKD (Dialysis)**	4 (3.6%)
**Immunosuppressed**	2 (1.8%)
**Infective Endocarditis**	3 (2.8%)
**Prior MI**	22 (20%)
**Atrial Fibrillation or Flutter**	36 (33%)
**Pacemaker**	9 (8.2%)
**Previous ICD**	2 (1.8%)
**Previous PCI**	37 (34%)
**Prior CABG**	10 (9.2%)
**Prior Aortic Valve replacement**	21 (19%)
**NYHA class III/IV symptoms**	89 (82%)
**Median STS Risk Score (IQR)**	4.4 (4.3)

CABG: coronary artery bypass grafting, ESKD: end-stage kidney disease, ICD: implantable cardioverter defibrillator, MI: myocardial infarction, NYHA: New York Heart Association symptom class, PCI: percutaneous coronary intervention, STS: Society of Thoracic Surgical risk score.

**Table 2:** Aortic stenosis subtypes, echocardiographic findings and procedural details.

Group Characteristics	n (%)
**Total patients**	109 (100%)
**AS Classification**	
Normal Severe AS	77 (71%)
Paradoxical LF LG AS	12 (11%)
Classical LF LG AS	9 (8.3%)
Low EF Normal Gradient AS	11 (10%)
**Median Aortic Valve Area (IQR)**	0.7 (0.2)
**Mean Stroke Volume Index (SD)**	38 (10)
**Median LVOT Diameter (IQR)**	2.0 (0.2)
**Median LVOT Velocity Time Integral (IQR)**	23 (9.0)
**Median BSA (IQR)**	1.8 (0.3)
**Median Aortic Peak Velocity (IQR)**	4.1 (0.6)
**Median Aortic Peak Gradient (IQR)**	70 (19)
**Procedural details**	
**Valve Type**	
Self-expanding valve (Medtronic Evolut R Pro/Pro+, Minneapolis, MN, USA)	63 (58%)
23 mm	4 (3.7%)
26 mm	21 (19%)
29 mm	27 (25%)
31 mm	1 (0.9%)
34 mm	10 (9.2%)
Balloon expandable valve (Edwards Sapien 3/Ultra, Edwards Lifesciences, Irvine, CA, USA)	46 (42%)
20 mm	1 (0.9%)
23 mm	17 (16%)
26 mm	20 (18%)
29 mm	8 (7.3%)
**Anesthesia Type**	
Moderate sedation	84 (77%)
General Anesthesia	25 (23%)

BSA: Body surface area; LF LG AS: Low flow, low-gradient aortic stenosis; LVOT: left ventricular outflow tract.

**Table 3:** Symptoms and echocardiographic parameters pre- and 30 days post-TAVR.

Group Characteristics	Pre-TAVR	Post-TAVR	
**Total patients**	109 (100%)	109 (100%)	*p*-value
**Median LA Strain at End Diastole (IQR)**			
Reservoir	18 (14)	22 (14)	<0.0001
Conduit	7.8 (4.8)	8.9 (5.4)	0.002
Contractile	9.6 (11)	12 (11)	0.003
**Median LA Strain at Onset of Atrial Contraction (IQR)**			
Reservoir	17 (11)	19 (11)	<0.0001
Conduit	7.0 (4.0)	8.2 (4.8)	0.01
Contractile	8.8 (9.2)	11 (9.1)	0.004
**Median Left Atrial Volume Index (IQR)**	47 (19)	46 (25)	0.48
**Mean LV GLS (SD)**	15 (3.5)	18 (3.3)	<0.0001
**Median LV Ejection Fraction (IQR)**	62 (13)	62 (6.0)	0.17
**Mean Aortic Valve Gradient (IQR)**	44 (16)	9.0 (7.0)	<0.0001
**Median KCCQ12 Score (IQR)**	34 (25)	79 (33)	<0.0001
**NYHA Class III/IV**	89 (82%)	12 (11%)	<0.0001

LA: left atrium, LV: left ventricle, LV GLS: left ventricular global longitudinal strain, KCCQ12: Kansas City Cardiomyopathy symptom score, NYHA: New York Heart Association symptom class.

**Table 4:** Symptoms and echocardiographic parameters 30 days post TAVR, according to AS subtype.

Group Characteristics	Normal Severe AS	Paradoxical LF LG AS	Classical LF LG AS	Low EF Normal Gradient AS	Paradoxical LF LG vs. Normal Severe AS	Classical LF LG vs. Normal Severe AS	Low EF Normal Gradient vs. Normal Severe AS	Normal EF AS Groups vs. Low EF AS Groups
**Total patients**	77	12	9	11	*p*-value	*p*-value	*p*-value	*p*-value
**Median % Change in LA Strain at End Diastole (IQR)**								
Reservoir	+19% (35%)	+5.6% (26%)	+29% (60%)	+6.2% (53%)	0.30	0.72	0.82	0.65
Conduit	+15% (66%)	+8.2% (59%)	+22% (47%)	+33% (82%)	0.51	0.61	0.59	0.80
Contractile	+18% (108%)	−2.4% (77%)	+68% (296%)	+14% (211%)	0.33	0.46	0.71	0.34
**Median % Change in LA Strain at Onset of Atrial Contraction (IQR)**								
Reservoir	+12% (34%)	+7.0% (20%)	+25% (56%)	+6.3% (54%)	0.34	0.76	0.82	0.66
Conduit	+13% (73%)	+9.3% (60%)	+8.9% (48%)	+29% (75%)	0.53	0.55	0.58	0.85
Contractile	+14% (102%)	−1.4% (66%)	+65% (255%)	+13% (189%)	0.33	0.44	0.72	0.33
**Median % Change in Left Atrial Volume Index (IQR)**	−4.6% (43%)	−4.5% (32%)	+8.3% (35%)	−12% (36%)	0.20	0.77	0.17	0.63
**Median % Change in LV GLS (IQR)**	+14% (25%)	+21% (24%)	+30% (42%)	+21% (36%)	0.5	0.35	0.21	0.16
**Median % Change in LV Ejection Fraction (IQR)**	0% (16%)	−0.8% (20%)	+17% (30%)	+31% (78%)	0.68	0.01	0.02	0.0002
**Median % Change in Mean Gradient (IQR)**	−77% (14%)	−68% (22%)	−75% (20%)	−85% (8.3%)	0.04	0.43	0.03	0.28
**Median KCCQ12 Score Post-Op (IQR)**	81 (31)	90 (41)	69 (28)	71 (20)	0.54	0.99	0.61	0.41
**Median Improvement in KCCQ12 Score Post-Op (IQR)**	+41 (33)	+54 (34)	+36 (22)	+35 (41)	0.48	0.43	0.25	0.07
**NYHA Class III/IV Post-Op**	9 (12%)	1 (8.3%)	1 (11%)	1 (9.1%)	1	1	1	0.69

KCCQ12: Kansas City Cardiomyopathy symptom score, NYHA: New York Heart Association symptom class.

**Table 5:** Baseline echocardiographic and clinical parameters according to heart failure hospitalization or death at 12 months.

Group Characteristics (Pre-TAVR)	No HF Hospitalization or Death at 12 Months	HF Hospitalization or Death at 12 Months	
**Total patients**	101 (92.3%)	8 (7.3%)	*p*-value
**Median LA Strain at End Diastole (IQR)**			
Reservoir	19 (14)	14 (10)	0.3
Conduit	8 (5)	7 (2)	0.5
Contractile	10 (11)	7 (9)	0.3
**Median LA Strain at Onset of Atrial Contraction (IQR)**			
Reservoir	17 (11)	13 (9)	0.3
Conduit	7 (5)	6 (1)	0.6
Contractile	9 (10)	6 (8)	0.3
**Median Left Atrial Volume Index (IQR)**	47 (19)	53 (26)	0.5
**Mean LV GLS (SD)**	15 (3.5)	15 (3.3)	0.7
**Median LV Ejection Fraction (IQR)**	62 (13)	58 (22)	0.3
**Mean Aortic Valve Gradient (IQR)**	43 (13)	41 (13)	0.7
**Median KCCQ12 Score (IQR)**	81 (33)	67 (25)	0.1

LA: left atrium, LV: left ventricle, LV GLS: left ventricular global longitudinal strain, KCCQ12: Kansas City Cardiomyopathy symptom score.

**Table 6:** Change in echocardiographic parameters at 30 days post-TAVR, and clinical events at 12 months.

Group Characteristics (Median % Change 30 Days Post TAVR)	No HF Hospitalization or death at 12 months	HF hospitalization or death at 12 months	
**Total patients**	101 (92.3%)	8 (7.3%)	*p*-value
**Median LA Strain at End Diastole (IQR)**			
Reservoir	14 (36)	−6 (69)	0.2
Conduit	16 (65)	−10 (85)	0.2
Contractile	19 (126)	11 (70)	0.2
**Median LA Strain at Onset of Atrial Contraction (IQR)**			
Reservoir	11 (33)	−6 (64)	0.2
Conduit	13 (71)	−13 (88)	0.2
Contractile	16 (109)	10 (68)	0.2
**Median Left Atrial Volume Index (IQR)**	−6 (38)	17 (29)	0.1
**Median LV GLS (IQR)**	16 (32)	20 (20)	0.6
**Median LV Ejection Fraction (IQR)**	0 (19)	−1.1 (23)	0.3

LA: left atrium, LV: left ventricle, LV GLS: left ventricular global longitudinal strain.

## Supplementary Materials

The following supporting information can be downloaded at: https://www.mdpi.com/article/10.3390/jcdd9020035/s1, Table S1: Symptoms and change in echocardiographic findings, post TAVR according to valve type.
