# Supplementary material for: The Effect of TAVR on Left Ventricular and Left Atrial Mechanics in Patients with Aortic Stenosis
Source: J Cardiovasc Dev Dis. 2022 Jan 21;9(2):35. doi: 10.3390/jcdd9020035 (PMC8877352; doi:10.3390/jcdd9020035)
Supplement: Supplementary file 1 [file jcdd-09-00035-s001.zip › jcdd-1550643-supplementary.pdf]

Table S1. Symptoms and change in echocardiographic findings, post TAVR according to valve type.

Group Characteristics	Balloon Expandable Valve (Edwards Sapien 3/Ultra)	Self-Expandable Valve (Medtronic Evolut R Pro)	
**Total patients**	46 (100%)	62 (100%)	*p*-value
**Median % Change in LA Strain at End Diastole (IQR)**			
Reservoir	+11% (36%)	+13% (35%)	0.7
Conduit	+7.0% (79%)	+22% (57%)	0.3
Contractile	+12% (94%)	+20% (119%)	0.4
**Median % Change in LA Strain at Onset of Atrial Contraction (IQR)**			
Reservoir	+7.8% (36%)	+11% (30%)	0.7
Conduit	+5.9% (77%)	+16% (59%)	0.3
Contractile	+10% (88%)	+18% (103%)	0.4
**Median % Change in Left Atrial Volume Index (IQR)**	−1.8% (32%)	−7.1% (41%)	0.8
**Median % Change in LV GLS (IQR)**	+17% (24%)	+16% (32%)	0.9
**Median % Change in LV Ejection Fraction (IQR)**	+4.4% (20%)	+0% (18%)	0.7
**Median % Change in Mean Aortic Gradient (IQR)**	−72% (18%)	−83% (12%)	**<0.01**
**NYHA Class III/IV Post-Op**	3(7.5%)	9 (16%)	0.4
